# Supplementary material for: An experimental test of the effects of redacting grant applicant identifiers on peer review outcomes
Source: eLife. 2021 Oct 19;10:e71368. doi: 10.7554/eLife.71368 (PMC8612703; doi:10.7554/eLife.71368)
Supplement: Supplementary file 1. — (A) Sample size calculations. (B) Number of matched white applications per matching criteria. (C) Redacted fields and their locations. (D) Differences between standard National Institutes of Health (NIH) review and this study. [file elife-71368-supp1.docx]

**Supplementary File 1**

**Supplementary File 1A**

**Table.** **Sample Sizes for Interaction Detection.** Sample size calculations to detect interactions in the primary analysis mixed-effects linear model.

| **Effect Size** | **ICC** | **Sample Size per Cell for Power 70%** | **Sample Size per Cell for Power 80%** | **Sample Size per Cell for Power 90%** |
| --- | --- | --- | --- | --- |
| 0.2 | 0.2 | 370 | 471 | 630 |
|  | 0.3 | 401 | 510 | 683 |
|  | 0.4 | 432 | 549 | 736 |
|  | 0.5 | 463 | 589 | 788 |
| 0.25 | 0.2 | 237 | 301 | 403 |
|  | 0.3 | 257 | 327 | 437 |
|  | 0.4 | 277 | 352 | 471 |
|  | 0.5 | 296 | 377 | 504 |
| 0.3 | 0.2 | 165 | 209 | 280 |
|  | 0.3 | 178 | 227 | 304 |
|  | 0.4 | 192 | 244 | 327 |
|  | 0.5 | 206 | 262 | 350 |

Notes:

1. ICC represents Intraclass Correlation Coefficient.
2. It reads, for example using row 3, with ICC = 0.3 and k = 2, a total of 1,604 (= 401*4) applications (or 401 applications per cell) is needed for power of 70% to detect an interaction effect of 0.2.

**Supplementary File 1B**

**Table. Number of matched white applications per matching criteria**

| **Number of Matched Variables** | **Number of Matched White Applications** |
| --- | --- |
| Matched all 8 criteria | 317 |
| Matched all 8 criteria  Excluding Degree Category | 53 |
| Matched all 8 criteria  Excluding Degree Category and Institution Rank Bin by NIH Dollars | 19 |
| Matched 7 criteria | 11 |

**Supplementary File 1C**

**Table. Redacted Fields and Their Application Locations**

| **Redacted Field** | **Location of Information** |
| --- | --- |
| **Application** | |
| **Principal Investigator name** | - Cover Page - Header - Research and Related Senior/Key Person Profile - Biographical Sketches - Research and Related Budget - Research Strategy - Multiple PI Leadership Plan |
| **Co-investigator names** | - Cover Page - Research and Related Senior/Key Person Profile - Research and Related Budget - Research Strategy - Multiple PI Leadership Plan |
| **Investigator title/position**  ***If faculty, redact rank and position (e.g., Assistant Professor and Professor both are completely redacted)** | - Cover Page - Possibly throughout (e.g., Biosketches) |
| **Employer Identification Number** | - Cover Page |
| **Type of applicant** | - Cover Page |
| **New investigator/Early stage investigator status** | - Cover Page |
| **Investigator profile (e.g., degree year, contact information, Congressional District)** | - Cover Page - Research and Related Senior/Key Person Profile - Biographical Sketch |
| **Institutional/organizational affiliation and location** | - Cover Page - Project/Performance Site Locations - Facilities and Other Resources - Research and Related Senior/Key Person Profile - Biographical Sketch - Research and Related Budget - Specific Aims - Research Strategy - Consortium/Contractual |
| **Project site location (name, address or specification of state, county, or region of country)** | - Project//Performance Site Locations - Project Summary/Abstract - Facilities and Other Resources - Research and Related Budget - Specific Aims - Research Strategy - Inclusion of Women and Minorities - Inclusion of Children |
| **Historic designation of research performance site** | - Research and Related Other Project Information |
| **Accession, funding opportunity, DUNS, and tracking numbers** | - Cover page - Project/Performance Site Locations - Research and Related Budget - Footer |
| **Human Subject Assurance Number** | - Research and Related Other Project Information |
| **PI and Investigators’ Personal Statement: Positions, Honors, and Service*; Selected Publications; Research Support***  ***Only information that identifies investigator or institution** | - Biographical Sketches |
| **Description of institutional/professional partnerships* (e.g., names of partners)**  ***Retaining information that helps reviewer assess adequate support** | - Specific Aims - Research Strategy - Multiple PI Leadership Plan - Consortium/Contractual - Data Sharing Plan |
| **Investigator’s past research/projects* (e.g., pronouns in descriptions that help identify, references to institutions)**  ***Retaining information that helps reviewer assess expertise of investigator** | - Specific Aims - Research Strategy - Multiple PI Leadership Plan |
| **References** | - Specific Aims - References Cited |
| **Letters of Support** | - Appended material |
| **Summary Statement** | |
| **Principal Investigator name(s)** | - Cover Page - Header - Critiques |
| **Co-investigator names** | - Cover Page |
| **Application number** | - Cover Page - Header |
| **Program contact** | - Cover Page |
| **Project site location** | - Critiques |
| **Institutional affiliation and professional partnerships (e.g., names of partners)** | - Critiques |
| **Study Section information** | - Meeting Roster |

**Supplementary File 1D**

**Table. Differences between standard NIH review and this study**

|  | **Standard NIH review** | **Anonymization Study Review** |
| --- | --- | --- |
| **Scientific Scope and Management of Peer Review** | Interactive peer review in 163 CSR study sections, managed by 163 CSR SROs | Non-interactive (mail) peer review process managed by 9 SSI contractor SROs. |
| **SRO Experience** | The average CSR SRO has 5 years of experience. New SROs are overseen by experienced supervisory SROs. | 9 SSI contractor SROs (3 retired CSR SROs, 1 former CSR contract SRO, and 5 with previous experience conducting federal peer review activities) |
| **SRO Training/Resources** | CSR SROs receive extensive training, including a 10-week course and regular training and mentoring by supervisors. SROs have access to the full range of NIH database resources to identify potential reviewers. | SSI contractor SROs were provided 6-hours of training and ad hoc guidance by an experienced CSR SRO. SSI contractor SROs did not have access to the NIH database resources. |
| **Reviewer identification and recruitment** | SROs examine the scientific content of the entire set of applications and identify/recruit expert reviewers based on a variety of resources including databases, publication records, funding history, suggestions by program staff or SRO colleagues, etc. SROs identify 3 reviewers for each application and assignments are made with the goal of ensuring both specific technical and broader expertise. | SSI SROs were provided the names and contact information of 19000 reviewers who had served on the study sections where 1200 applications had been reviewed. They developed key words for each application and matched these with the weighted RCDC terms of the reviewers, to determine whether the scientific expertise was a match. Contractor SROs in the study had to identify 6 reviewers for each application (3 for standard and 3 for redacted format). |
| **Reviewer training** | SROs conduct pre-meeting training teleconferences with reviewers, walk them through policy updates, dos and don’ts of critique preparation, scoring, discussion | Reviewers were sent guidelines and critique templates. |
| **Reviewer attrition** | Infrequent | High |
| **Preliminary score guidance** | R01-specific NIH score chart for overall impact, pre-meeting SRO/reviewer training on scoring procedures.  Assigned reviewers are able to calibrate their preliminary overall impact scores prior to meeting during the read phase; thus, preliminary overall impact scores can change. | Reviewers were provided general NIH score chart used for criteria scores or other, non-R01 mechanisms as well as standard teaching materials to score R01 applications in meeting materials.  Assigned reviewers did not have the opportunity to calibrate their preliminary overall impact scores. |
| **Review meeting and discussion among reviewers** | Yes | No |
| **Final scores** | Yes | No |
