# Supplementary material for: An experimental test of the effects of redacting grant applicant identifiers on peer review outcomes
Source: eLife. 2021 Oct 19;10:e71368. doi: 10.7554/eLife.71368 (PMC8612703; doi:10.7554/eLife.71368)
Supplement: Supplementary file 2. [file elife-71368-supp2.docx]

review Critique Template and questionNaire

**RPG/X01/R01/R03/R21/R33/R34 Review**

If you cannot access the hyperlinks below,

visit <http://grants.nih.gov/grants/peer/critiques/rpg_D.htm>.

Application #:

Principal Investigator(s):

**Overall Impact**

Reviewers will provide an overall impact score to reflect their assessment of the likelihood for the project to exert a sustained, powerful influence on the research field(s) involved, in consideration of the following five scored review criteria, and additional review criteria. An application does not need to be strong in all categories to be judged likely to have major scientific impact.

| [Overall Impact](http://grants.nih.gov/grants/peer/critiques/rpg_D.htm#rpg_overall) *Write a paragraph summarizing the factors that informed your Overall Impact score.* |
| --- |
|  |

**Scored Review Criteria**

Reviewers will consider each of the five review criteria below in the determination of scientific and technical merit, and give a separate score for each.

| 1. [Significance](http://grants.nih.gov/grants/peer/critiques/rpg_D.htm#rpg_01) |
| --- |
| **Strengths**  **Weaknesses** |

| 2. [Investigator(s)](http://grants.nih.gov/grants/peer/critiques/rpg_D.htm#rpg_02) |
| --- |
| **Strengths**  **Weaknesses** |

| 3. [Innovation](http://grants.nih.gov/grants/peer/critiques/rpg_D.htm#rpg_03) |
| --- |
| **Strengths**      **Weaknesses** |

| 4. [Approach](http://grants.nih.gov/grants/peer/critiques/rpg_D.htm#rpg_04) |
| --- |
| **Strengths**      **Weaknesses** |

| 5. [Environment](http://grants.nih.gov/grants/peer/critiques/rpg_D.htm#rpg_05) |
| --- |
| **Strengths**      **Weaknesses** |

**Additional Review Criteria**

As applicable for the project proposed, reviewers will consider the following additional items in the determination of scientific and technical merit, but will not give separate scores for these items**.**

- Responses for Protections for Human Subjects, Vertebrate Animals, and Biohazards **are required from reviewers for all applications**.

| A response for Inclusion of Women, Minorities and Children **is required from reviewers** for applications proposing Human Subjects Research.[Protections for Human Subjects](http://grants.nih.gov/grants/peer/critiques/rpg_D.htm#rpg_humans) |
| --- |
| Comments (Required Unless Not Applicable):      Data and Safety Monitoring Plan (Applicable for Clinical Trials Only):  Comments (Required Unless Not Applicable): |

| [Inclusion of Women, Minorities and Children](http://grants.nih.gov/grants/peer/critiques/rpg_D.htm#rpg_inclusion) **Applicable Only for Human Subjects research and not IRB Exemption #4.** |
| --- |
| - Sex/Gender: - Race/Ethnicity: - For NIH-Defined Phase III trials, Plans for valid design and analysis: - Inclusion/Exclusion of Children under 18:   Comments (Required Unless Not Applicable): |

| [Vertebrate Animals](http://grants.nih.gov/grants/peer/critiques/rpg_D.htm#rpg_animals) |
| --- |
| Is the proposed research involving vertebrate animals scientifically appropriate, including the justification for animal usage and protections for research animals described in the Vertebrate Animal section?  Comments (Required Unless Not Applicable): |

| [Biohazards](http://grants.nih.gov/grants/peer/critiques/rpg_D.htm#rpg_biohazards) |
| --- |
| Comments (Required Unless Not Applicable): |

| [Resubmission](http://grants.nih.gov/grants/peer/critiques/rpg_D.htm#rpg_resubmission) |
| --- |
| Comments (if applicable): |

| [Renewal](http://grants.nih.gov/grants/peer/critiques/rpg_D.htm#rpg_renewal) |
| --- |
| Comments (if applicable): |

| [Revision](http://grants.nih.gov/grants/peer/critiques/rpg_D.htm#rpg_revision) |
| --- |
| Comments (if applicable): |

**Additional Review Considerations**

**As applicable** for the project proposed, reviewers will address each of the following items, but will not give scores for these items and should not consider them in providing an overall impact/priority score.

| [Applications from Foreign Organizations](http://grants.nih.gov/grants/peer/critiques/rpg_D.htm#rpg_foreign) |
| --- |
| Comments (Required Unless Not Applicable): |

| [Select Agents](http://grants.nih.gov/grants/peer/critiques/rpg_D.htm#rpg_agents) |
| --- |
| Comments (Required if Unacceptable): |

| [Resource Sharing Plans](http://grants.nih.gov/grants/peer/critiques/rpg_D.htm#rpg_sharing) |
| --- |
| Comments (Required if Unacceptable): |

| [Authentication of Key Biological and/or Chemical Resources](http://grants.nih.gov/grants/peer/critiques/rpg_D.htm#rpg_authentication) |
| --- |
| Comments (Required if Unacceptable): |

| [Budget and Period of Support](http://grants.nih.gov/grants/peer/critiques/rpg_D.htm#rpg_budget) |
| --- |
| Recommended budget modifications or possible overlap identified: |

**Additional Comments to Applicant**

Reviewers may provide guidance to the applicant or recommend against resubmission without fundamental revision.

| [Additional Comments to Applicant](http://grants.nih.gov/grants/peer/critiques/rpg_D.htm#rpg_additional) (Optional) |
| --- |
|  |

**MORE QUESTIONS FOLLOW ON THE NEXT PAGE**

**PLEASE COMPLETE THESE QUESTIONS AFTER YOU HAVE COMPLETED YOUR EVALUATION OF THE APPLICATION. THANK YOU.**

| 1. **Based on what you have read, please make your best judgments and indicate your levels of confidence:** |
| --- |
| **Based on what you have read, please make your best judgment as to** **GENDER of this applicant.**  **Are you confident with this decision?**    **Based on what you have read, please make your best judgment as to** **ETHNICITY of this applicant.**    **Are you confident with this decision?**    **Based on what you have read, please make your best judgment as to** **RACE of this applicant.**    **Are you confident with this decision?**    **Based on what you have read, please make your best judgment as to** **INSTITUTIONAL AFFILIATION of this applicant.**    **Are you confident with this decision?**  From which institution/lab do you think this application was submitted?    From which investigator or investigative group do you think this application was submitted?    **MORE QUESTIONS FOLLOW ON THE NEXT PAGE**    **Based on what you have read, please make your best judgment as to** **CAREER STAGE of this applicant.**    **Are you confident with this decision?**   1. **Based on what I read, I feel this application:**  - Is interesting and convincing with a well-defined research hypothesis.      - Demonstrates how the proposed work will advance the mission of NIH, field, or improve clinical practice.      - Clearly articulates the scope of the project (not too broad or narrow), and shows consistency among the aims, methods, and analysis plan.      - Is clearly organized, well-written, and easy to follow.      - Enabled me to generate informed conclusions about the proposed project.      1. **I believe that reviewers can provide a fair, thorough, and competent review if applications are anonymized.**     **YOU HAVE COMPLETED ALL QUESTIONS** |
